# Associations between energy intake, daily food intake and energy density of foods and BMI *z*-score in 2–9-year-old European children

**DOI:** 10.1007/s00394-013-0575-x

**Published:** 2013-09-06

**Authors:** A. Hebestreit, C. Börnhorst, G. Barba, A. Siani, I. Huybrechts, G. Tognon, G. Eiben, L. A. Moreno, J. M. Fernández Alvira, H. M. Loit, E. Kovacs, M. Tornaritis, V. Krogh

**Affiliations:** 1 Department of Epidemiological Methods and Etiologic Research, Leibniz Institute for Prevention Research and Epidemiology, BIPS GmbH, Achterstrasse 30, 28359 Bremen, Germany; 2 Institute of Food Sciences, National Research Council, Avellino, Italy; 3 Department of Public Health, Ghent University, Ghent, Belgium; 4 Department of Public Health and Community Medicine, University of Gothenburg, Göteborg, Sweden; 5 GENUD (Growth, Exercise, Nutrition and Development) Research Group, School of Health Sciences, University of Zaragoza, Zaragoza, Spain; 6 Department of Chronic Diseases, National Institute for Health Development, Tallinn, Estonia; 7 Department of Pediatrics, University of Pécs, Pecs, Hungary; 8 Research and Education Institute of Child Health, Strovolos, Cyprus; 9 Department of Preventive and Predictive Medicine, Fondazione IRCCS Istituto Nazionale dei Tumori, Milan, Italy; 10 Dietary Exposure Assessment Groups, International Agency for Research on Cancer, Lyon, France

**Keywords:** Energy intake, Daily food intake, Energy density of foods, BMI *z*-score, Children

## Abstract

**Purpose:**

The aim of this study was to investigate the associations between proxy-reported energy intake, daily food intake and energy density of foods and body mass index (BMI) *z*-score in 2–9-year-old European children.

**Methods:**

From 16,225 children who participated in the identification and prevention of dietary- and lifestyle-induced health effects in children and infants (IDEFICS) baseline examination, 9,782 children with 24-h proxy dietary information and complete covariate information were included in the analysis. Participating children were classified according to adapted Goldberg cutoffs: underreports, plausible energy reports and overreports. Energy intake, daily food intake and energy density of foods excluding noncaloric beverages were calculated for all eating occasions. Effect of energy intake, daily food intake and energy density of foods on BMI *z*-score was investigated using multilevel regression models in the full sample and subsample of plausible energy reports. Exposure variables were included separately; daily food intake and energy intake were addressed in a combined model to check for interactions.

**Results:**

In the group of plausible energy reports (*N* = 8,544), energy intake and daily food intake were significantly positively associated with BMI *z*-score. Energy density of foods was not associated with BMI *z*-score. In the model including energy intake, food intake and an interaction term, only energy intake showed a significantly positive effect on BMI *z*-score. In the full sample (*N* = 9,782), only energy intake was significantly but negatively associated with BMI *z*-score.

**Conclusion:**

Proxy-reporters are subject to misreporting, especially for children in the higher BMI levels. Energy intake is a more important predictor of unhealthy weight development in children than daily food intake.

## Introduction

The prevalence of childhood overweight and obesity worldwide is increasing dramatically [1] and is a growing public health concern. The rise in childhood overweight and obesity is a likely consequence of the modern obesogenic and predominantly automated environment characterized by growing sedentary activities and less physical activity [2]. Besides lack of sleep [3] and longer screen time [4], altered dietary behavior toward more frequent breakfast skipping [5], eating out [6], constant eating [7] and high energy snacking [8, 9] and less family meals or fixed meal times [10, 11] seems to enhance the problem.

Portion sizes consisting mainly of high-energy-dense foods instead of water-containing foods such as fruits and vegetables have increased over the past decades [12]. The portion size (or the amount of eaten food in gram) is highly interrelated with energy intake (in kilocalories, kcal) and energy density of foods [the energy content per amount of food (kcal/g)]. The energy density of foods of a given amount of food (portion size) decreases with increasing water content, since water adds weight and not energy [13]. Energy intake is associated with larger portion sizes and has been shown to increase the risk of excess body weight [14, 15].

Due to the dramatic development of childhood obesity, this subject has of late been focused on in numerous studies conducted worldwide. Energy intake, daily food intake and energy density of foods have been linked to the obesity epidemic, mainly in studies carried out in the USA and in some European countries. To date, no study has investigated this association in young children under free-living conditions in a multinational study, following a standardized study protocol [16].

The present study aims to add new information to observed associations between energy intake, daily food intake and energy density of foods and body mass index (BMI) *z*-score of young populations across Europe. The association between the exposure variables and BMI *z*-score was investigated in a sample that included (a) potentially misreported energy intakes and (b) only plausible reported energy intakes based on a 24-h dietary recall.

## Participants and methods

### Study population

Analyses were based on data of the IDEFICS (identification and prevention of dietary- and lifestyle- induced health effects in children and infants) study, which is the largest European multicenter study to date, aiming to investigate the causes and consequences of overweight and obesity in 2- to 9-year-old children. The IDEFICS baseline survey enrolled 16,864 children (response rate 53.4 %), 16,225 (51.4 %) of whom fulfilled the inclusion criteria (complete information on age, sex, height and weight). The baseline survey was conducted in eight European countries (Sweden, Germany, Hungary, Italy, Cyprus, Spain, Belgium and Estonia) from September 2007 to May 2008. Sampling and basic study characteristics have been described elsewhere [17].

In each country, participating centers obtained ethical approval from the local responsible authorities in accordance with the ethical standards laid down in the 1964 Declaration of Helsinki and its later amendments. All children gave personal assent, and the parents or custodian of the child provided written informed consent for all examinations and the collection of samples, analysis and storage of personal data and collected samples. Several modules including interviews with parents and questionnaires pertaining to lifestyle habits and dietary intakes, as well as anthropometric measurements, were incorporated in the survey [18].

### Anthropometric measurements

The field methods for assessing weight (kg) and height (cm) comprised anthropometric measurements for standing height using a Seca 225 stadiometer (Seca GmbH & KG) according to international standards for anthropometric assessment [19]. Body weight was assessed in fasting children using a prototype of the TANITA BC 420 SMA digital scale (TANITA Europe GmbH, Sindelfingen, Germany) specifically adapted for children’s feet. All measurements were performed in light clothing (e.g., underwear). BMI was calculated by dividing body weight in kilograms by squared body height in meters and then transformed to an age- and sex-specific *z*-score. BMI *z*-score is a measure of relative weight adjusted for child age and sex, as BMI for children cannot be interpreted as in adults for children (the cutoffs of 25 and 30 for overweight and obesity, respectively, are not applicable for children as these are age and sex dependent). BMI *z*-scores and weight groups (overweight/obese and thin/normal) were defined using age- and sex-specific cutoff values according to Cole et al. [20, 21].

### The computer-based 24-h dietary recalls and school meal assessments

In IDEFICS, dietary intake of the previous 24 h was assessed using ‘Self-Administered Children and Infant Nutrition Assessment’ (SACINA) [22]. The type and amount of all foods and drinks consumed during the previous day, starting with the first meal, snack or drink after waking up in the morning; and school or preschool meals, drinks and snacks consumed the day prior to the 24-h dietary recall (24HDR) were assessed using a standardized observer sheet, which was completed by trained personnel [23]. The software was based on the ‘Young adolescents’ nutrition assessment on computer’ (YANA-C) system [24] developed within the HELENA study (http://www.helenastudy.com). It was structured according to six meal occasions: breakfast, mid-morning snack, lunch, afternoon snack, evening meal and evening snack. In SACINA, standardized photographs were available to assist portion size estimation. The IDEFICS study protocol required the assessment of one 24HDR in all children and repeated 24HDR interviews in a convenience sample. In the present study sample, 23 % of the recalls were weekend days (Friday, Saturday and Sunday), and 77 % working days (Monday to Thursday).

Country-specific food composition tables (FCT) were used to match simple foods or pan-European homogeneous multi-ingredient food items [25–29]. Estonia combined the Norwegian and Finnish FCT [30, 31], while Cyprus included foods from the German and Swedish FCT. For harmonization, all energy and nutrient data of the country-specific FCT were expressed in 100 g standard portion per food ‘as consumed.’ Standard units were taken from McCance and Widdowson’s [32]. Finally, total energy content was calculated in kcal.

The proxies, mainly the parents, were assisted by trained survey personnel or the dietician from the survey team when completing the 24HDR. The required time frame for one interview was 20–30 min [22].

The validity of proxy-reported energy intake from the 24HDR was tested using the doubly labeled water technique in young children. The instrument was found to be valid to assess energy intake on group level [33].

### Data cleaning

Multiple steps of data cleaning assured data quality since not only recall and reporting bias, but also erroneous data entry, incorrect coding and false standard amounts could have resulted in missing and implausible data. Missing quantities or implausible values (above median + 2.5 standard deviation or intakes of >1,500 ml (gastric capacity) [34] for single food items) that could not be corrected were imputed by country, food group and age-specific median intakes (0.01 % of the entries). Incomplete 24HDR and 24HDR with four or more imputed values were excluded from the analysis. From the full survey sample of 16,225 children, subjects with one complete 24HDR and covariate information required were included in the study final analysis (*N* = 9,782). No significant differences were observed between survey and study sample characteristics such as age, gender, weight status, educational level of parents and energy reporting.

### Data analysis

Consistency of proxy-reported energy intake with energy requirements was estimated using the ratio of proxy-reported energy intake over predicted basal metabolic rate [35]. This was calculated to classify all individuals with underreported energy intake, plausible reported energy intake and overreported energy intake according to adapted Goldberg cutoffs [36]. Goldberg cutoff values were recalculated for application in children [37] using age- and sex-specific reference values.

The following eating habit parameters were investigated: total daily energy intake (1 unit ~ 100 kcal of total daily intake), total daily food intake and drinks (1 unit ~ 100 g of total daily food intake) and total energy intake (kcal/day) divided by total daily food intake (g/day). Parameters were calculated in 100 g or 100 kcal units since we did not expect relevant effects on BMI *z*-score of 1 g or 1 kcal units. Daily food intake and energy density of foods were calculated excluding noncaloric beverages, such as (table, mineral, natural) water, plain (herbal) tea and (surrogate) coffee and (carbonated) beverages with artificial sweeteners.

The correlation between exposure variables (energy intake, daily food intake and energy density of foods) was tested in the full sample and in the subsample of plausible energy proxy-reports only.

The effect of the total energy intake, daily food intake and energy density of foods on BMI *z*-score was investigated using multilevel regression models (SAS: PROC MIXED). To account for cluster effects, a random effect was added for the study center. Exposure variables were investigated in separate models where all models were run including all children (*N* = 9,782) and including only children whose 24HDR were classified as plausible proxy-reports (*N* = 8,544). Since energy intake and daily food intake are highly interrelated, their predictive power on BMI *z*-score was tested in a combined model including an additional interaction term.

All models were adjusted for age and sex of the child and maximum educational level of both parents according to International Standard Classification of Education (ISCED). ISCED level was used as proxy indicator for socioeconomic status (SES) of the family. To account for clustered study design, a random effect was added for the study center. Statistical significance was set at *P* ≤ 0.01. All statistical analyses were performed using SAS 9.2 (SAS Institute, Cary, NC, USA).

## Results

### Descriptive statistics

Approximately 21 % of the study population was overweight or obese, with a higher prevalence among school children (26 %) than preschool children (15 %) (Table 1). The study sample included the highest proportion (20 %) of dietary data from Italian children and the lowest (4 %) from Belgian children. In total, 87 % (*N* = 8,544) of the 24HDRs were classified as plausible energy reports.

**Table 1:** Descriptive characteristics of the study population (total group and stratified by age; total numbers and percentages)

	Children 2–<6 years		Children 6–<10 years		All	
	*N*	%	*N*	%	*N*	%
*Energy reporting* ^*a*^						
Overreports	163	3.7	149	2.7	312	3.2
Plausible energy reports	3,948	90.7	4,596	84.6	8,544	87.3
Underreports	241	5.5	685	12.6	926	9.5
*Study center*						
Italy	854	19.6	1,086	20.0	1,940	19.8
Estonia	700	16.1	602	11.1	1,302	13.3
Cyprus	410	9.4	663	12.2	1,073	11.0
Belgium	225	5.2	158	2.9	383	3.9
Sweden	574	13.2	639	11.8	1,213	12.4
Germany	735	16.9	1,017	18.7	1,752	17.9
Hungary	544	12.5	980	18.0	1,524	15.6
Spain	310	7.1	285	5.2	595	6.1
*ISCED*-*level* ^*b*^						
Primary education	121	2.8	167	3.1	288	2.9
Lower secondary education	392	9.0	525	9.7	917	9.4
(Upper) secondary education	1,532	35.2	1,926	35.5	3,458	35.4
Postsecondary, nontertiary education	766	17.6	909	16.7	1,675	17.1
First stage of tertiary education	1,541	35.4	1,903	35.0	3,444	35.2
*Weight status* ^*c*^						
Thin/normal weight	3,704	85.1	4,038	74.4	7,742	79.1
Overweight/obese	648	14.9	1,392	25.6	2,040	20.9
*Sex of the child*						
Male	2,245	51.6	2,679	49.3	4,924	50.3
Female	2,107	48.4	2,751	50.7	4,858	49.7

^a^Proxy-reporting classification in underreport, plausible report and overreport according to adapted Goldberg cutoffs (Bornhorst et al. [37])

^b^Maximum of both parents

^c^Weight categories according to Cole et al. [20]

 The mean daily energy intake was 1,511 kcal in the full sample (*N* = 9,782) and 1,547 kcal in the plausible energy reporting subsample (Table 2). The average daily food intake (excluding noncaloric beverages) was 1,213 g in the full sample and 1,247 g in the plausible energy reporting subsample. The daily energy density of foods was 1.3 kcal/g in both samples. In the plausible energy reports, subsample mean daily energy intake was 1,520 kcal among the thin/normal weight children and 1,660 kcal among overweight/obese children. The mean daily food intake among thin/normal weight children and overweight/obese children was 1,251 g and 1,231 g, respectively. Among thin/normal weight children and overweight/obese children, the mean energy density of foods was 1.3 and 1.4, respectively.

**Table 2:** Dietary characteristics of the full study sample and subsample of plausible reporters (*N*, means and standard deviations)

	Full study sample (*N* = 9,782)						Plausible energy reporters (*N* = 8,544)		
	Energy reporting^a^			Weight status			Weight status		
	Energy overreporters *N* = 312	Plausible energy reporters *N* = 8,544	Energy underreporters *N* = 926	Thin/normal weight *N* = 7,742	Overweight/obese *N* = 2,040	All *N* = 9,782	Thin/normal weight *N* = 6,884	Overweight/obese *N* = 1,660	All *N* = 8,544
	Mean ± std	Mean ± std	Mean ± std	Mean ± std	Mean ± std	Mean ± std	Mean ± std	Mean ± std	Mean ± std
Daily energy intake (kcal)	2,714 ± 420	1,547 ± 418	772 ± 235	1,502 ± 506	1,546 ± 532	1,511 ± 512	1,520 ± 408	1,660 ± 438	1,547 ± 418
Daily food intake (g)	1,779 ± 431	1,247 ± 404	709 ± 293	1,227 ± 437	1,160 ± 439	1,213 ± 438	1,251 ± 401	1,231 ± 414	1,247 ± 404
Energy density of foods (kcal/g)	1.6 ± 0.4	1.3 ± 0.4	1.2 ± 0.5	1.3 ± 0.4	1.4 ± 0.5	1.3 ± 0.4	1.3 ± 0.4	1.4 ± 0.5	1.3 ± 0.4

^a^Proxy-reporting classification according to adapted Goldberg cutoffs (Bornhorst et al. [37])

Exposure variables (energy intake, daily food intake and energy density of foods) were significantly correlated in the plausible proxy-reports subsample (*P* < 0.0001): Daily energy intake was directly correlated with daily food intake (*r* = 0.58) and energy density of foods (*r* = 0.25); energy density of foods was inversely correlated with daily food intake (*r* = −0.58).

#### Results of the multilevel regression models

Overall, we investigated the influence of daily intake of foods (g/day), energy intake (kcal/day) and energy density of foods (kcal/g) on the BMI *z*-scores of 9,782 children (Table 3, models labeled with ‘a’ relate to the full study sample). In the combined model 4a, energy intake was significantly negatively associated with BMI *z*-score. The other exposure variables were not associated with BMI *z*-score in the full study sample (models 1a, 2a, 3a). In the plausible energy reports subsample (*N* = 8,544, Table 3, models labeled with ‘b’), energy intake (model 1b) and daily food intake (model 2b) were significantly positively associated with BMI *z*-score. In the combined model 4b, energy intake was significantly positively associated with BMI *z*-score.

**Table 3:** Associations between energy intake, daily food intake and energy density of foods and BMI *z*-score adjusted for age, sex and ISCED level and including study center as random effect

Full sample (*N* = 9,782)				Plausible energy reports (*N* = 8,544)			
Parameter	Estimate	Standard Error	*P* value	Parameter	Estimate	Standard error	*P* value
*Model 1a* ^*a*^				*Model 1b* ^*b*^			
Intercept	−0.560	0.137	0.004	Intercept	−0.878	0.132	0.0003
Daily energy intake (1 unit ~ 100 kcal)	−0.002	0.003	0.427	Daily energy intake (1 unit ~ 100 kcal)	0.032	0.004	<0.0001
*Model 2a* ^*a*^				*Model 2b* ^*b*^			
Intercept	−0.623	0.138	0.003	Intercept	−0.794	0.147	0.001
Daily food intake (1 unit ~ 100 g)	0.0037	0.0033	0.297	Daily food intake (1 unit ~ 100 g)	0.027	0.004	<0.0001
*Model 3a*				*Model 3b* ^*b*^			
Intercept	−0.520	0.143	0.008	Intercept	−0.555	0.139	0.005
Energy density of foods (kcal/g)	−0.056	0.037	0.131	Energy density of foods (kcal/g)	0.042	0.040	0.302
*Model 4a* ^*a*^				*Model 4b* ^*b*^			
Intercept	−0.450	0.167	0.031	Intercept	−0.956	0.195	0.002
Daily energy intake (1 unit ~ 100 kcal)	−0.0191	0.007	0.007	Daily energy intake (1 unit ~ 100 kcal)	0.030	0.010	0.002
Daily food intake (1 unit ~ 100 g)	−0.002	0.009	0.839	Daily food intake (1 unit ~ 100 g)	0.012	0.013	0.321
Energy density of foods (kcal/g)	0.001	0.001	0.076	Energy density of foods (kcal/g)	−0.0002	0.001	0.740

Models 1–3: association between exposure variables and BMI *z*-score was investigated in separate models

Model 4: association between exposure variables and BMI *z*-score was investigated in a combined model including an additional interaction term

^a^Effects of the dietary variables in the full sample

^b^Effects of the dietary variables in the subsample of plausible energy reports

In all models, age of the child and low education level of the parents were significantly and directly positively associated with BMI *z*-score (data not shown).

## Discussion

Energy intake is associated with larger portion sizes and has been proven to increase the risk of unhealthy weight development [14, 15]. In the plausible energy reports subsample, energy intake and daily food intake were positively associated with BMI *z*-score in regression analysis. When energy intake, food intake and an interaction term were combined in the same model, only energy intake remained positively associated with BMI *z*-score in children. This finding indicates that a large amount of foods facilitates the overconsumption of energy but is not independently associated with BMI *z*-scores in children. Hence, for the prevention of obesity in children, health education should focus on age-appropriate energy intake and portion sizes [38]. Even the reduction in item size of (snack) foods has been found to decrease consumption and may be an effective strategy to reduce obesity-related eating patterns, such as the easy availability of large food portions [39]. However, reverse causality has to be taken into account when interpreting cross-sectional associations. The validity of future analyses of the IDEFICS longitudinal data will possibly be less affected by such bias.

Energy intake may be influenced by the energy density (kcal/g) of foods and beverages [13]. The calculation of energy density of foods strongly differs between studies and can be based on all foods consumed. Drinking water or even all kind of beverages is generally excluded. In the present study, daily food intake and energy density of foods included foods and energy-containing beverages since satiation studies have shown that humans tend to consume a constant amount of foods by weight or volume on a day-to-day basis [13]. Although an association between energy density of foods, excluding beverages and water, and body weight was observed in very recent publications [40, 41], we did not exclude energy-containing beverages since they contribute increasingly to the daily energy intake, e.g., sugar-sweetened soft drinks and milks [42, 43]. We ran additional models based on the food only method for energy density calculation with similar results: Energy density showed no association with BMI *z*-score in the full sample or in the plausible energy reports subsample (data not shown).

High-energy-dense diets were found to be associated with obesity in children. This was linked to greater intakes of energy, fat and added sugars, and to significantly lower intake of fruits and vegetables [44]. Children from low SES families were found to have an increased energy intake from larger portions of energy-dense foods, while consuming large portions of low-energy-dense vegetables was associated with lower energy intake [45]. A recent systematic literature review found moderately strong evidence from longitudinal studies of a positive association between dietary energy density and increased childhood obesity [46]. In the present study, energy density of foods was not found to be associated with BMI *z*-score. The inverse correlation of energy density of foods with daily food intake, however, showed that under free-living conditions, children seem to adjust their daily food intake in relation to energy density of foods.

In the IDEFICS study, proxies—mainly the parents—completed the 24HDR for the children. Since proxy-reporting relates to the number of meals under parental control, inaccurate estimation and incomplete reports of food and beverage consumption may also contribute toward reporting bias [47]. Thus, one important barrier for the completeness of food reporting in the IDEFICS study was the consumption of foods without parental control, for example meals and beverages consumed in school and/or kindergarten. The latter could not be recalled and entered in SACINA by the parents. The IDEFICS study reduced this problem by using trained personnel to collect dietary information during school time for the day prior to the 24HDR [22]. Even though we were able to capture school meals and school snacks, our results indicate a certain degree of misreporting through missing dietary information for snacking or out of home meals especially in the full sample and in the higher BMI levels. Not only overweight/obese children give biased information on macronutrients [48], food intake [49] and energy intake [50], their parents also tend to misreport food and nutrient intakes [51, 52] such as unhealthy foods high in fat and simple carbohydrates, especially when these foods relate to obesity. The present study supports the prevailing opinion that self-reporters are subject to bias and that misreporting is one of the main errors in dietary assessment. Although multiple 24HDRs were available from certain centers, only the first complete 24HDR was included in the present analysis in order to achieve adequate statistical power for a cross-country analysis. The inclusion of repeated recalls may have helped to account for within-person variation, but this would not have solved the problem of (energy) misreporting. Individuals who tend to misreport on the first recall day are likely to do so when completing additional recalls.

The accurate estimation of portion sizes also poses a challenge to untrained interviewees. Photographs in automated 24HDR have been shown to be useful for accurate portion size estimation among adults [53], although both underestimation [54] and overestimation [55] of the portion size have been reported in other studies. In SACINA, quantities were mainly assessed by photographs of serving sizes, standard portions, customary packing size and foods in pieces or slices in order to reduce reporting bias [22]. However, inaccuracy of portion size estimation cannot be entirely ruled out.

The participation proportion of 53.4 % may appear to be low, and we have no systematic information on nonparticipants. However, due to the community-oriented and setting-based study design, the IDEFICS study approached the whole population for participation, and proportions of sexes and education-level characteristics of study participants are comparable to the general population. By taking this into consideration and by excluding subjects with implausible energy reports from the study sample, we assume no selection effects on the study outcomes.

Excess body weight is the result of an imbalance between energy intake and total energy expenditure [56]. Physical activity may therefore confound associations between eating habit variables and BMI *z*-score. Hence, we adjusted for proxy-reported physical activity (total daily duration of outdoor playing in minutes) in preliminary analyses (data not shown). The validity of proxy-reported physical activity data was, however, considered questionable as it was positively associated with the BMI *z*-score in all regression models. The estimate for the exposure variables changed only marginally while the significance remained unchanged; thus, we decided not to include the physical activity variable in the regression models. In general, validity and reliability of proxy-reported physical activity have been observed to be low for questionnaire surveys in youth [57].

An issue which has been controversially discussed in the literature is the possible influence of eating frequency on total daily energy intake. Even though some studies observed that a high number of eating episodes are inversely associated with body fat or obesity in children [58, 59], it has also been discussed that constant eating is a strong predictor for overeating and overweight [7]. The SACINA program offered six meal occasions for dietary reporting. If children consumed additional snacks or drinks intermediately, proxies added such foods and beverages to the predetermined meal occasions. The association between constant eating or meal/snack portion sizes and BMI *z*-score was therefore difficult to estimate.

Nevertheless, the IDEFICS study permits a novel deeper insight into dietary behavior of 2–9-year-old children across Europe. The large sample size comprising data from eight European countries, the strictly standardized data assessment, documentation and data cleaning processing, guarantee the highest possible data quality.

## Conclusion

The IDEFICS study is the first study to investigate the association between proxy-reported daily food intake, energy intake and energy density of foods and BMI *z*-score in 2–9-year-old European children. The results indicate that proxy-reporters are subject to misreporting especially for children in the higher BMI levels and that energy intake is a more important predictor of unhealthy weight development in children than daily food intake. The promotion of age-appropriate energy intake should therefore be emphasized in the prevention of overweight among children.

We advise that future investigations focus on the effects of energy intake and energy density of foods on weight development, preferably in a longitudinal data set.
